# Integrating AI in medical education: embracing ethical usage and critical understanding

**DOI:** 10.3389/fmed.2023.1279707

**Published:** 2023-10-13

**Authors:** Faiza Alam, Mei Ann Lim, Ihsan Nazurah Zulkipli

**Affiliations:** Pengiran Anak Puteri Rashidah Sa'adatul Bolkiah Institute of Health Sciences, Universiti Brunei Darussalam, Bandar Seri Begawan, Brunei

**Keywords:** generative AI, medical higher education, critical thinking, ethical considerations, healthcare professionals

## Introduction

Artificial intelligence, commonly abbreviated as AI, is described as the simulation of human intelligence by machines, giving machines, particularly computer systems, the ability to perform complex tasks that would normally require human reasoning, decision-making, and problem-solving. With advancements in machine learning and deep learning, we can expect to see AI integrated into even more areas of our daily lives. Indeed, AI is becoming increasingly prevalent in healthcare. It can provide personalized recommendations (1) and improve medical diagnostics, particularly in the field of medical imaging (2). At least 11 medical schools across the United States of America, Canada and the Republic of Korea have pilot AI curricula (3). Potentially, all medical schools may need to do the same to keep up with not only the changing landscape of medical education but indeed of medicine itself.

The AI chatbot ChatGPT took the world by storm in late 2022, and higher education institutions are not immune from its repercussions in education. Online education platforms have already started to use generative AI tools, such as Khan Academy via the AI chatbot, Khanmingo, to supplement and personalize student education. It can be used as a personal tutor, a writing coach, and a question generator to test comprehension. It is only a matter of time before brick-and-mortar institutions need to do the same to keep up with the rapidly changing landscape of digital tools in education. Furthermore, Clark and Archibald (4) asserted that the avoidance of the impact of generative AI on higher education results in multi-level harms which range from the lack of structures to ensure scholarly integrity to the stagnation and irrelevance of learning approaches in health science education.

Despite the acceptance of AI in the healthcare profession, concerns such as legal liability and accountability have been raised at the crux of the profession, that is, in making clinical decisions (5). At present, it remains unclear to healthcare professionals who is responsible in the event of errors by AI in clinical decision support systems. Also, it appears that many would not assume accountability for results provided by AI (6). The ethical sense when using AI can be fostered by educators during medical education to facilitate accountable and responsible AI usage by future healthcare professionals.

AI tools in medical education can encompass AI virtual environments, AI-based assessments, and adaptive e-learning systems (7). Additionally, three distinct uses of AI tools in education have been defined in a report by Baker and Smith (8), listed below:

1. Learner-oriented AI—AI technology that “students use to receive and understand new information”.
2. Instructor-oriented AI—AI technology that “can help teachers reduce their workload, gain insights about students and innovate in their classroom”.
3. Institution-oriented AI—AI technology that “helps make or inform decisions made by those managing and administrating schools or our education system as a whole”.

As healthcare education faculty members in a higher education institution, we advocate for the integration of AI tools in medical education, specifically learner-oriented AI tools in this paper. In this article, we focus on learner-oriented tools and propose guidelines for which these tools should be used by medical students.

## Impact of AI use in an undergraduate medical program

With the release of ChatGPT late last year, efforts have been made to investigate its utility in education, as well as the utility of other generative AI tools (9–12). However, because of its capabilities, ChatGPT is easily misused, and we outline an observation from our faculty below.

At the Pengiran Anak Puteri Rashidah Sa'adatul Bolkiah (PAPRBS) Institute of Health Sciences at Universiti Brunei Darussalam, undergraduate students of the medicine program are introduced to academic writing as part of the mandatory research module in the curriculum. The module is designed to equip students with the fundamentals to carry out research in science, medicine, and health. For examination, students are expected to report findings scientifically in the form of a written manuscript and oral presentation. Manuscripts are submitted online via the Canvas learning management system and are checked for plagiarism using Turnitin (13).

AI writing had been detected across several pieces of submission, including heavy incorporation of ChatGPT content that gave rise to serious concerns of academic offense. There was evidence of citations to papers which do not exist (an AI phenomenon known as “hallucination”), and the writing was found “nicely summarized generally”, but the knowledge was “touched on superficially and not critically”. The lack of knowledge depth and critical thinking was also demonstrated during the oral presentation.

In summary, generative AI is a powerful tool and its usage by students is currently creating disconcerting experiences in higher education due to its impact on academic integrity.

## Training students and faculty staff for ethical use of AI

Our experience above indicates to us that faculty teaching staff needs to engage with AI in the interest of training medical students and keeping the curriculum current. Students may plagiarize essays from content generated by AI tools, compromising their critical thinking skills and scholarly integrity. To address this, universities should implement strict policies and guidelines for the ethical use of AI applications. They should foster a culture of academic integrity and equip students with the necessary skills to critically evaluate and use AI responsibly. Educators can also provide workshops or training sessions to teach students how to effectively use AI tools, balancing their use with their own unique ideas and voices. This approach promotes originality, independent thinking and accountability alongside technological advancement, and supports the interest and positive attitude that faculty teaching staff and medical students have toward AI (14).

In the aforementioned undergraduate research module, we propose guiding medical students on how to write effective ChatGPT prompts and use ChatGPT answers to avoid ChatGPT downsides (such as hallucination and phrase inaccuracies), while maintaining academic integrity and writing standards. During the introductory briefing at the start of the module, we strongly encourage our medical educators who are presenting to remind students of two Turnitin features which detect plagiarism—similarity and AI scores. Medical educators and students should be aware that software programs exist to detect AI-generative text and should take measures to avoid plagiarism (15). To promote accountability, we should require students to disclose the use of AI tools in their written manuscript in a manner similar to how we disclose our use of statistical software for data analysis (15, 16).

Elsewhere in the curriculum, AI-related lectures could be introduced to first-year medical students, in which the topics cover basic understanding, benefits and risks of AI, which would allow students to become familiar with common terminology (3, 17). In modules which emphasize biomedical knowledge acquisition and retention, as well as those related to patient care, teaching staff can incorporate large language model-powered chatbots to answer common medical questions for students and patients. Chatbots can also be used by students to practice their clinical communication skills.

Apart from the integration of AI tools in the curriculum, we further propose AI-based research projects to be made available for medical students on the research project and dissertation track of the fourth year of the curriculum at PAPRSB Institute Health Sciences. This proposal is feasible based on collaborations between our medical school and the School of Digital Sciences on campus.

Scientists have tried to use ChatGPT to write scientific manuscripts with varying success (12, 18), while Yu and Guo (19) elaborated on issues to be resolved when using generative AI in education, which we have summarized below together with potential solutions (Table 1). Ethical issues arising from the use of AI tools such as legal liability and accountability can be discussed in the research module as well as in the medical ethics module in the second year of the curriculum.

**Table 1:** Ethical issues of generative AI and their potential solutions (19).

**Issue**	**Solution**
Opacity and inexplicability	Develop interpretable generative AI algorithms that explain the AI decision-making process
Data privacy and security	Use of encryption technology, access control mechanisms and data anonymization, and de-identification
Personalization and fairness	Develop algorithms that can identify potentially unfair factors and eliminate them while still meeting the needs of the students
Effectiveness and reliability	Improve quality and quantity of data by including diverse data, but still ensuring quality and consistency of labeled data

## Beneficial and ethical usage of AI in medical education

Through our conversations with medical students, we can affirm the common usage of ChatGPT. AI writing tools (such as ChatGPT and QuillBot) are prevalent and readily accessible to students right now, and these can enhance research capabilities, as ChatGPT did for college-educated professionals in improving the quality and enjoyment of their writing tasks (20). For medical students, ChatGPT can be used to provide interactive environments for them to practice their communication skills with simulated patients and another tool, DALL-E, can be used to help them practice their diagnostic imaging skills (9).

The ethics of using AI tools is indeed a prevalent concern today. Medical students need to be guided to think independently and appraise critically as well as ethically despite the convenience of readily generated information. They need to be aware of and steer clear of AI-generated misinformation on biomedical knowledge and the pathophysiology of disease (3, 11), and thereby avoid AI-generated information acting as a crutch for clinical decision-making and hampering clinical reasoning abilities (17).

In summary, toward embracing AI education in the medical curriculum and in keeping the curriculum relevant to ongoing development in AI, faculty teaching staff need to rapidly adapt to the integration of AI tools and develop AI literacy by being trained in multiple AI tools for teaching and assessment.

## Conclusion

AI has emerged as a powerful tool in medical education, offering unique opportunities to enhance learning, address knowledge gaps, and improve patient care. Embracing AI in medical education holds immense potential for advancing healthcare and empowering the next generation of future healthcare professionals to enhance patient care, improve outcomes, and contribute to the advancement of medicine. By leveraging the capabilities of AI, medical education can become more personalized and efficient. However, careful attention must be paid to ethical considerations, technical infrastructure, and faculty training to ensure the responsible integration of AI into medical education. Medical education should include dedicated modules or courses that explore the ethical considerations of AI and emphasize the need for transparency and comprehensibility in AI systems to foster trust between students, faculty, and AI technologies.

## Author contributions

INZ: Conceptualization, Writing—original draft, review, and editing. FA: Conceptualization, Writing—original draft. MAL: Conceptualization, Writing—original draft, review, and editing.
